# The Effects of 12 Weeks of Concurrent and Combined Training on Inflammatory Markers, Muscular Performance, and Body Composition in Middle-Aged Overweight and Obese Males

**DOI:** 10.3390/nu15061482

**Published:** 2023-03-20

**Authors:** Reza Bagheri, Mehdi Kargarfard, Khosro Jalali, Damoon Ashtary-Larky, Neda Cheraghloo, Hamid Ghobadi, Babak Hooshmand Moghadam, Alexei Wong, Michael Nordvall, Frédéric Dutheil

**Affiliations:** 1 Department of Exercise Physiology, University of Isfahan, Isfahan 8174673441, Iran; 2 Sports Physiology Department, Islamic Azad University, Isfahan Branch (Khorasgan), Isfahan P.O. Box 81595-158, Iran; 3 Nutrition and Metabolic Diseases Research Center, Ahvaz Jundishapur University of Medical Sciences, Ahvaz 61357-1579, Iran; 4 Department of Epidemiology and Biostatistics, School of Public Health, Tehran University of Medical Sciences, Tehran 1417613151, Iran; 5 Department of Exercise Physiology, Ferdowsi University of Mashhad, Mashhad 9177948974, Iran; 6 Department of Health and Human Performance, Marymount University, Arlington, VA 22207, USA; 7 Université Clermont Auvergne, CNRS, LaPSCo, Physiological and Psychosocial Stress, CHU Clermont-Ferrand, University Hospital of Clermont-Ferrand, Preventive and Occupational Medicine, Witty Fit, F-63000 Clermont-Ferrand, France

**Keywords:** exercise, obesity, metabolic syndrome, inflammation

## Abstract

Aim: Previous studies have focused on the order of endurance and resistance training when performing concurrent training (CT). However, no study has compared the effects of combined training with CT orders on inflammatory markers, muscular performance, and body composition in overweight and obese males. Therefore, the purpose of the current study was to compare the effects of 12 weeks of CT and combined training on the aforementioned markers in overweight and obese males. Methods: Sixty middle-aged overweight and obese males (age 51 ± 4 years) were randomly assigned into one of four groups: endurance followed by resistance training (ER; *n* = 15), resistance followed by endurance training (RE; *n* = 15), combined resistance and endurance training (COM), or control (CON; *n* = 15). Anthropometric, body composition, inflammatory marker, and muscular performance measurements were collected at baseline and after 12 weeks. Results: FFM remained unchanged in all three intervention groups (*p* > 0.05). Reductions in FM in the RE group were significantly greater than in CON (*p* = 0.038). The increases in serum concentrations of adiponectin in the RE group were significantly greater than in all other groups (*p* < 0.05). Increased serum concentrations of CTRP3 in all intervention groups were significantly greater than the CON group (*p* < 0.05); moreover, the increases in the RE group were significantly greater than CON (*p* < 0.001). Regarding CTRP5, the increase in RE was significantly greater than COM (*p* = 0.014). The RE group experienced significantly greater increases in CTRP9 than all other groups (*p* < 0.05), and the decreases in serum concentrations of CRP and TNF-α were significantly greater in the RE group compared to CON and ER (*p* < 0.05). Vo_2max_ in the ER group was significantly greater than COM (*p* = 0.009), and all interventions resulted in higher gains compared to CON (*p* < 0.05). The increases in leg press strength, chest press strength, lower-body power, and upper-body power in the RE group were significantly greater than in the COM group (*p* < 0.05). In addition, the increases in chest press strength in the ER group were significantly greater than COM (*p* = 0.023). Conclusions: Regardless of training order, CT improved inflammatory markers, body composition, power, and VO_2max_. Notably, our analysis indicated significantly greater improvements in adiponectin, CTRP5, CTRP9, CRP, and TNF-α levels when RT preceded ET in CT sessions compared to other exercise training sequences. These findings suggested that the order of exercise training may have a significant impact on the effectiveness of CT on inflammatory markers, which has potential implications for exercise prescription and optimization of health-related training outcomes.

## 1. Introduction

Obesity is linked to chronic low-grade inflammation, which can lead to metabolic dysfunction and related diseases such as coronary artery disease (CAD) [1,2]. Adipose tissue produces adipokines, which can have pro-inflammatory or anti-inflammatory effects [3]. Dysregulation of adipokine synthesis or release may contribute to the development of obesity-related disorders [1,4].

Adiponectin and members of the C1q/tumor necrosis factor-related protein (CTRP) family are peptides secreted by adipocytes that regulate metabolism and inflammation [5]. CTRP family members, which have structural similarities with adiponectin [6], might play a significant role in the development and progression of CAD. The primary CTRP family members connected to the pathophysiology of CAD are CTRP3, CTRP5, and CTRP9. These proteins primarily operate in white adipose tissue around the heart and to a lesser extent in visceral tissue such as the liver, governing endothelial function, inflammatory response, and metabolic dysfunction [7,8].

CTRP3 is a cardioprotective and anti-inflammatory cytokine found in human plasma [9]. By modulating inhibitory toll-like receptors (TLRs) and nuclear factor kappa B (NF-κB) signaling, CTRP3 reduces insulin resistance and obesity-related chronic inflammation [10]. After coronary stent insertion, elevated levels of CTRP5 may cause immediate restenosis by promoting the growth, migration, and inflammation of vascular smooth muscle cells through activating the Notch1, transforming growth factor (TGF)-β, and hedgehog pathways [11]. CTRP9, the closest paralog of adiponectin, plays a role in controlling lipid metabolism by demonstrating anti-inflammatory and anti-atherosclerotic properties and providing cardioprotective benefits in CAD. Additionally, CTRP9 activates the Akt, AMPK, and p42/44 MAPK pathways to enhance glucose uptake, thereby regulating glucose metabolism [12].

Compelling epidemiological evidence supports the hypothesis that regular exercise training, including endurance training (ET) and resistance training (RT), confer therapeutic and preventive benefits by counteracting degenerative processes associated with obesity. Such benefits encompass a reduction in systemic inflammatory markers as well as weight loss and salubrious effects for individuals with a variety of cancer types [13,14]. Accordingly, exercise, particularly RT, has become increasingly popular. These advantages align with the latest guidelines from both the World Health Organization and the American College of Sports Medicine, which endorse physical activity as an intervention for enhancing overall health.

The anti-inflammatory benefits of regular exercise may be mediated by both a decrease in visceral fat mass (by lowering the production of adipokines) and the development of an anti-inflammatory milieu after exercise bouts [15]. ET and RT are frequently prescribed to reduce inflammatory-related disease risk [13]; however, incorporating RT and ET simultaneously in the same training period—also known as combined training or concurrent training (CT) [16,17]—may further enhance the anti-inflammatory benefits of regular exercise [18]. To our knowledge, a single study has measured biomarkers of the CTRP family after exercise. Hae Yoon Choi et al. demonstrated that a 3-month combined exercise program (45 min of aerobic exercise at an intensity of 60–75% of maximum heart rate and 20 min of resistance training performed five times per week) significantly decreased CTRP3 levels and modestly increased CTRP5 levels in obese Korean women [19], which contradicted evidence showing increased CTRP3 following physical activity. In multiple regression models, CTRP3 concentrations were favorably linked with adiponectin but adversely associated with retinol-binding protein 4 (RBP4) levels. RBP4 induces insulin resistance in adipocytes indirectly by increasing proinflammatory cytokine secretion from macrophages [19].

Despite the fact that CT may result in a number of advantageous adaptations, there are possible issues regarding whether some of the targeted adaptations to RT or ET that occur (depending on the order) could be compromised due to an “interference effect” [20]. The interference effect simply states that ET signaling stunts muscle growth or other muscular adaptations (e.g., muscular strength, power, etc.) [21]. There are insufficient data about the impact of exercise sequence within a session on interference effects between acute bouts of RT and ET on human performance and/or chronic health-related adaptations. Nevertheless, a rationale for RT and ET training orders during CT may be dependent on molecular interference effects, particularly when applied to special populations such as those with obesity. Due to the molecular signaling mechanisms involved in RT and ET, the sequence of RT and ET in a CT session (i.e., endurance followed by resistance exercise vs. resistance followed by endurance exercise) may yield distinct effects. Performing RT prior to ET, cycling instead of running, separating exercise bouts by 6–24 h, and adopting strategies that minimize overall exercise volume (i.e., utilizing high-intensity intervals, 2–3 days of ET, increasing protein intake, etc.) may potentially minimize reductions in muscular adaptations when concurrently performing ET and RT [21].

Other crucial factors associated with adaptation to exercise training, such as inflammatory markers, have not been investigated in the context of CT order. In the present study, we included a combined training group, which sometimes is used interchangeably with CT. In combined training, in contrast to CT, for instance, participants do not finish RT before transitioning to perform ET. However, participants perform six sets of RT and then move to perform 10 min of ET (combined training is outlined in Materials and Methods section). The main reason for the inclusion of this training protocol is that the anabolic environment of RT may reduce the interference effect of ET on muscular adaptations. Therefore, the primary this study aimed to investigate the effects of 12 weeks of CT order and combined training on inflammatory markers (adiponectin, tumor necrosis factor-α (TNF-α), C-reactive protein (CRP), CTRP3, CTRP5, and CTRP9) in overweight and obese males. We hypothesized that various orders of RT and ET would stimulate secretions of adiponectin, TNF-α, CRP, CTRP3, CTRP5, and CTRP9. The second objective was to investigate the impact of training procedures on body composition, muscular performance, and cardiorespiratory fitness.

## 2. Materials and Methods

### 2.1. Participants

Sixty overweight and obese middle-aged men (age: 51 ± 4 years) took part in this study. The inclusion criteria were: age ˃ 40 years, body mass index (BMI > 27 kg·m^−2^), sedentary (less than 1 h of activity per week in the previous year), 7–8 h of sleep per 24 h, and no chronic use of nutritional supplements or pharmaceuticals, particularly nonsteroidal anti-inflammatory drugs. In addition, participants were otherwise healthy and devoid of health complications including but not limited to Parkinson’s disease, heart disease, and diabetes. Further participant inclusion criteria requirements were non-smoking status and no hormonal or mental health therapies, no regular moderate-to-heavy aerobic or resistance training within the past year, and alcohol consumption. Supplementation and the usage of drugs that alter the metabolism of muscle mass or fat mass (FM) were also regarded as exclusion criteria. A physician examined all possible participants based on these criteria using the Physical Activity Readiness Questionnaire (PAR-Q) and medical health/history questionnaires. In order to mitigate attrition, study participants were informed that successful completion of their 12-week intervention with high adherence would entitle them to an additional 6 months of complimentary training and nutrition services following the conclusion of the study. The protocol for the research was approved by the Institutional Human Subjects Committee. The research procedures were conducted at the Azad University of Najadfabad in Isfahan, Iran (RECNAJAFABADIAUIR.1399.08), and all experiments adhered to the Declaration of Helsinki.

### 2.2. Study Design

**Figure 1:**
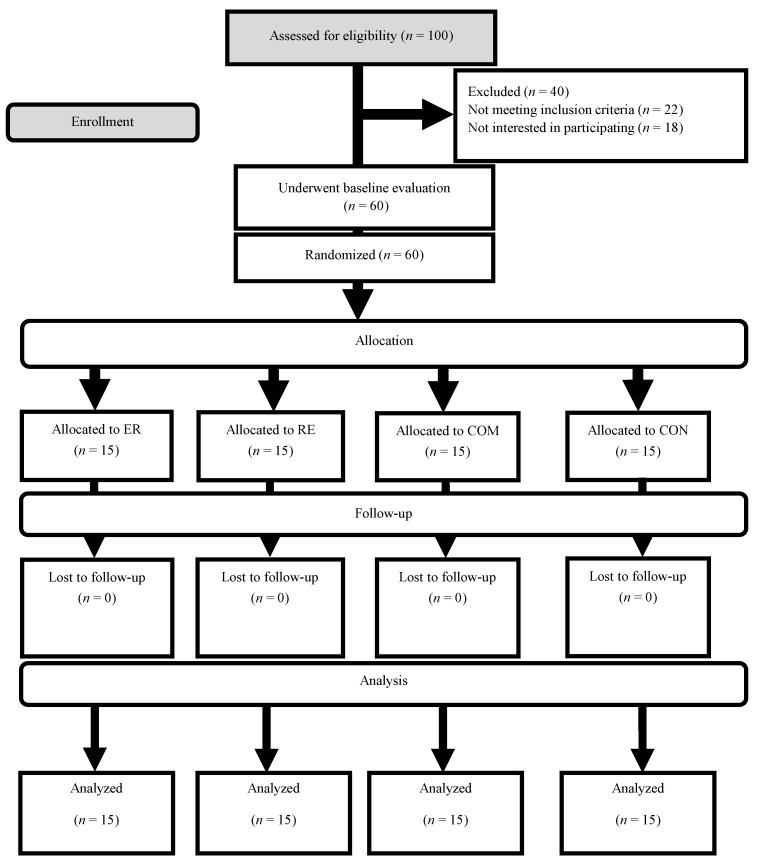
Flow of participant recruitment.

A participant eligibility and allocation flowchart is depicted in Figure 1. Before the initial measurements, all tests and methods were thoroughly explained to the participants, who were randomly assigned into one of four groups: endurance followed by resistance training (ER; *n* = 15), resistance followed by endurance training (RE; *n* = 15), combined resistance and endurance training (COM), or control (CON; *n* = 15). Anthropometric, body composition, inflammatory marker, and strength and aerobic performance measurements were collected at baseline and 12 weeks post-intervention (48–72 h after the last training session). Every measurement was taken at the same time of day (within 1 h) and under identical environmental conditions. For the course of the trial, participants were asked not to modify their typical lifestyle and food choices.

### 2.3. Anthropometry and Body Composition

Participants were instructed upon entering the laboratory to urinate (void) completely within 30 min of the test. Each participant’s body mass was measured using a digital scale (lumbar, Hong Kong, China) to the nearest 0.1 kg, and height was measured with a stadiometer (Race industrialization, Shanghai, China) to the nearest 0.1 cm. Bioelectrical impedance equipment (Inbody 720, Seoul, Republic of Korea) was used to conduct the BMI, waist–hip ratio (WHR), fat mass (FM), and fat-free mass (FFM) measurements. Before the test, the participants were told to abstain from physical activity for 48 h and fast for 12 h (overnight with at least 8 h of sleep).

### 2.4. Blood Sampling and Analysis

After a 12 h overnight fast, samples (5 mL) were collected from the cubital vein using standard techniques. Blood samples were collected at baseline and 48 h after the last training session. Following completion of blood collection, samples were centrifuged at 3000 rpm for 20 min, and the serum was stored at −70 °C for future analysis of adiponectin, CTRP3, CTRP5, CTRP9, TNF-α, and CRP (ZellBio GmbH, Lonsee, Germany).

### 2.5. Strength Testing

After a body composition evaluation and blood test, a strength test was conducted 24 h later. In order to calculate customized training intensity for the RT protocol, a one-repetition maximum (1RM) was calculated. Before commencing testing, research personnel discussed each test’s objective, associated risks, potential discomforts, and participant obligations. Prior to the testing phase, all participants were advised to abstain from consuming alcohol for 48 h, caffeinated beverages for 12 h, and meals for 2 h. Nevertheless, ad libitum water consumption was permitted. After a short general and specific warm-up, all exercises included in the RT program were evaluated for strength testing (i.e., leg extension, leg curl, bench press, lat pulldown, lateral raise, and abdominal crunch) using variable-resistance machines. The participants performed two attempts separated by a 5 min rest period, and their highest lifted weight and number of repetitions [22] were recorded. The number of repetitions to fatigue did not exceed 10. Maximal strength was estimated from these assessments using a previously published equation: 1RM = weight/(1.0278 − 0.0278 × reps) [21].

### 2.6. Power Testing

Upper- and lower-body anaerobic power was assessed via Monark Wingate cycle ergometry (Monark model 894e, Vansbro, Sweden) as previously described. Briefly, participants were acquainted with the test and instructed to stay seated in the saddle for the test duration. Participants cycled or cranked against a pre-determined resistance (7.5% of body mass for the lower body test and 5.5% for the upper body test) as fast as possible for 30 s. Participants were verbally encouraged to pedal as hard and fast as possible throughout the whole 30 s test. Peak power output was documented in real time during the test using Monark Anaerobic test software (3.3.0.0).

### 2.7. Aerobic Power

VO_2max_ was estimated using the Modified Bruce procedure. The treadmill began at 2.74 km per hour (1.7 miles per hour) and 0% grade (or incline). During three-minute intervals (stages), the treadmill’s elevation and/or speed increased. When participants were unable to continue due to exhaustion, discomfort, or other medical indicators as previously indicated, the test was terminated [23]. The following formula was utilized for VO_2max_ prediction: VO_2max_ = 14.8 − (1.379 × T) + (0.451 × T²) − (0.012 × T³), where T is the time in minutes to complete the test.

### 2.8. Concurrent Training

**Figure 2:**
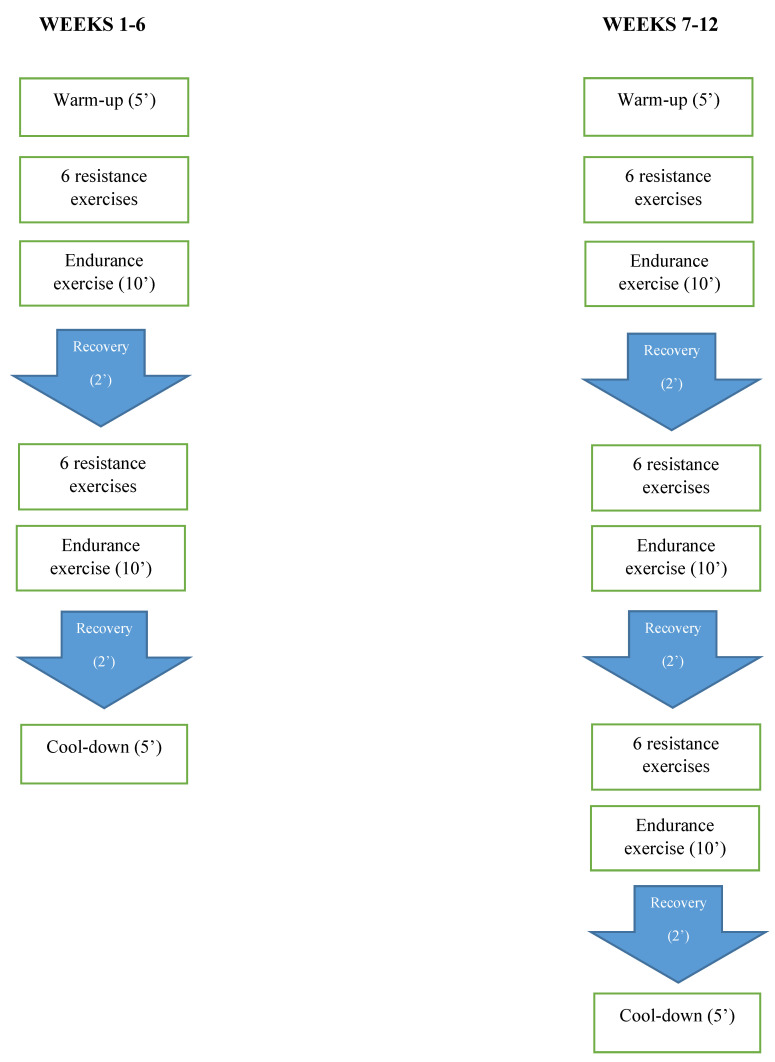
COM training protocol.

Participants in all training groups completed the supervised training 3 times a week, with at least 48 h between each session, for 12 weeks. The ER group performed ET first followed immediately by RT, while the RE group performed RT first followed immediately by ET. Participants in the COM group also performed both RT and ET protocols with a combination block of resistance and endurance exercises repeated twice in weeks 1–6 and three times in weeks 7–12 to achieve three sets per resistance exercise and 30 min of ET. The order of RT and ET in the COM group is reported in Figure 2.

### 2.9. Preparatory Phase

Prior to the intervention data collection, all participants completed one week of CT consisting of three exercise sessions to familiarize themselves with ET and RT. This phase was intended to provide education on proper lifting methods, familiarization with all exercises and equipment, and confirmation that all participants began the research with equivalent levels of expertise [24].

### 2.10. Resistance Training Protocol

Following the preparatory phase, RT was initiated at 50% of the 1RM and gradually increased to 80% of 1RM during the final week of the intervention. A total of 8 to 14 repetitions were performed over two sets during weeks 1 through 6 and three sets during weeks 7 through 12. Furthermore, rest intervals between sets ranged from 30 to 75 s and were progressively increased in correspondence with the intensity of the RT. The exercises utilized in the study included leg extension, leg curl, bench press, lat pulldown, lateral raise, and abdominal crunch performed on variable-resistance machines as noted above.

### 2.11. Endurance Training Protocol

The exercise regimen consisted of 20 min at 55% of maximum heart rate on a fixed-speed bicycle ergometer (11900 Community Rd, Poway, CA, USA) in the first week, which progressed to 30 min at 70% of maximal heart rate in the final week of the intervention. A polar heart rate monitor was used to measure the intensity of the workout (Polar S810, Polar Electro, Kempele, Finland).

### 2.12. Cool Down

Irrespective of group assignment, all participants completed a 10 min active cool-down consisting of low-intensity exercise on a cycle ergometer (4 min), slow walking (4 min), and light lower extremity static stretching with an emphasis on the quadriceps, hamstrings, gastrocnemius, and erector spinae.

### 2.13. Nutrient Intake and Dietary Analysis

Prior to training, participants were encouraged to record as precisely as possible, using 24 h food log recalls, every calorie and nutrient consumption ingested over the course of 6 days (4 non-consecutive weekdays and 2 non-consecutive weekend days). Dietary record information was deemed the participant’s usual diet, and participants were required to maintain this diet throughout the duration of the study. Food records were kept daily by participants throughout the study using the mobile phone applications Easy Diet Diary (Xyris Software Pty Ltd., Brisbane City, Australia) for those with iPhones (Apple Inc., Cupertino, CA, USA; *n* = 28) and My fitness pal (MyFitnessPal Inc., San Francisco, CA, USA), Iran, for those with Android-based devices (*n* = 32). After the entry of each food item into Diet Analysis Plus version 10 (Cengage, Boston, MA, USA), total energy consumption and the amount of energy generated from macronutrients (proteins, fats, and carbohydrates) were determined.

### 2.14. Statistical Analysis

An a priori sample size calculation was conducted using G-power 3.1.9.2 software. The rationale for the sample size was based on a previous work, which documented significant improvements in CRP concentrations in overweight and obese individuals. By utilizing the equation for effect size (ES) {(mean before − mean after CT)/the pooled standard deviation}, this study revealed an ES of 0.53 {(4.90 − 3.54)/5.07}. In the present study and based on α = 0.05, a power (1 − β) of 0.80 and an ES = 0.53 (highest approximate effect size), a total sample size of at least 44 participants (*n* = 11 per group) was needed for sufficient power to detect significant changes in CRP concentrations. We recruited 15 participants per group due to potential dropouts. All data and values are presented as means ± standard deviations (SDs). An analysis of variance (ANOVA) was used to compare the mean values for each variable between groups. Tukey’s honestly significant difference (HSD) test was used to determine if the difference in variables between two sets of groups was statistically significant. The Bonferroni test was used to compare the mean values between each pair of groups. The paired *t*-test was applied to compare the means of two variables for the same subject. An analysis of covariance (ANCOVA) was used to evaluate whether the means of variables in the post-test were equal across levels of each participant group, thus controlling the effects of variables in the pre-test. Pearson’s linear regressions were performed with a 95% confidence interval (CI). Training volume was analyzed using ANOVA with repeated measures. All analyses were performed using SPSS (version 26). *p*-values less than 0.05 were regarded as statistically significant. Figures were generated using GraphPad Prism (version 8.4.3).

## 3. Results

### 3.1. Body Composition

All pre- and post-intervention anthropometric and body composition data are shown in Table 1. All three intervention groups showed a significantly decreased body mass (RE = −5.6 kg (95% confidence interval = −3.3 to −7.9; *p* < 0.001; ES = 1.28), ER = −4.3 kg (95% CI = −2.1 to −6.5; *p* = 0.001; ES = 0.57), and COM = −3.3 kg (95% CI = −1.3 to −5.2; *p* = 0.003; ES = 0.78)) and BMI (RE = −1.9 kg·m^−2^ (95% CI = −1.1 to −2.7; *p* < 0.001; ES = 1.51), ER = −1.4 kg·m^−2^ (95% CI = −0.7 to −2.1; *p* = 0.001; ES = 0.99), and COM = −1.1 kg·m^−2^ (95% CI = −0.4 to −1.7; *p* = 0.003; ES = 0.28)), while FM was decreased only in the RE and ER groups (RE = −5.9 kg (95% CI = −1.3 to −10.5; *p* = 0.014; ES = 0.82) and ER = −4 kg (95% CI = −0.1 to −8; *p* = 0.044; ES = 0.62)). In addition, WHR significantly decreased only in the RE and COM groups (RE = −0.037 m (95% CI = −0.018 to −0.05; *p* = 0.001; ES = 0.23) and COM = −0.034 m (95% CI = −0.015 to −0.053; *p* = 0.044; ES = 1.47)). FFM remained unchanged in all three intervention groups (*p* > 0.05). The ANCOVA results showed that the changes in BM and BMI in RE were significantly greater than in the CON group. Changes in FM for participants in the RE group were significantly greater than in CON. The reductions in WHR were significantly greater in RE than in ER and CON; also, the decreases in COM were significantly greater than in ER and CON.

### 3.2. Inflammatory Markers

**Figure 3:**
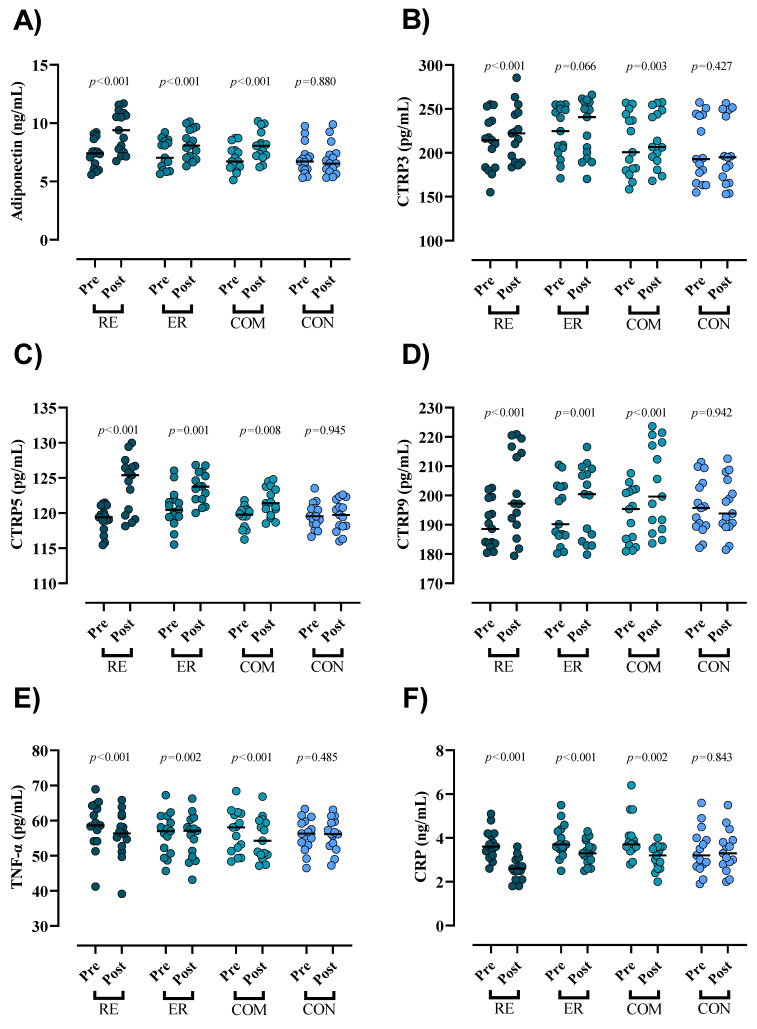
Effects of concurrent and combined training on changes in serum markers throughout the intervention: (**A**) adiponectin (ng/mL); (**B**) CTRP3 (pg/mL); (**C**) CTRP5 (pg/mL); (**D**) CTRP9 (pg/mL); (**E**) TNF-α (pg/mL); (**F**) CRP (ng/mL). *n* = 15 per group and *p*-values above time points indicate paired sample *t*-test results.

All pre- and post-intervention inflammatory marker results are shown in Figure 3. All three interventions showed significantly increased serum concentrations of adiponectin (RE = 1.9 ng/mL (95% CI = 2.3 to 1.5; *p* < 0.001; ES = 1.3), ER = 0.9 ng/mL (95% CI = 1 to 0.8; *p* < 0.001; ES = 0.75), and COM = 1 ng/mL (95% CI = 1.2 to 0.9; *p <* 0.001; ES = 0.91)); CTRP5 (RE = 5 pg/mL (95% CI = 6.4 to 3.5; *p* < 0.001; ES = 1.70), ER = 2.9 pg/mL (95% CI = 4.5 to 1.3; *p* = 0.001; ES = 1.21), and COM = 2.1 pg/mL (95% CI = 3.5 to 0.6; *p =* 0.008; ES = 1.17)); and CTRP9 (RE = 11.6 pg/mL (95% CI = 15.6 to 7.6; *p* < 0.001; ES = 1.02), ER = 4.1 pg/mL (95% CI = 6.4 to 1.9; *p* = 0.001; ES = 0.36), and COM = 9.6 pg/mL (95% CI = 12.7 to 6.4; *p <* 0.001; ES = 0.78)), while CTRP3 was significantly increased only in the RE and COM groups (RE = 10.9 pg/mL (95% CI = 15.8 to 6; *p* < 0.001; ES = 0.34) and COM = 6.3 pg/mL (95% CI = 10 to 2.5; *p* = 0.003; ES = 0.18)). However, all three intervention groups showed significantly decreased serum concentrations of TNF-α (RE = −2.6 ng/mL (95% CI = −1.8 to −3.4; *p* < 0.001; ES = 0.39), ER = −0.7 ng/mL (95% CI = −0.3 to −1.1; *p* = 0.002; ES = 0.11), and COM = −1.9 ng/mL (95% CI = −1 to −2.7; *p <* 0.001; ES = 0.31)) and CRP (RE = −1.1 ng/mL (95% CI = −0.8 to −1.3; *p* < 0.001; ES = 2.2), ER = −0.5 ng/mL (95% CI = −0.2 to −0.7; *p* < 0.001; ES = 0.78), and COM = −0.8 ng/mL (95% CI = −0.3 to −1.3; *p =* 0.002; ES = 1.15)). The ANCOVA results showed that the increases in serum concentrations of adiponectin in the RE group were significantly greater than in all other groups (*p* < 0.05). In addition, the increased adiponectin in all intervention groups was significantly greater than in the CON group (*p* < 0.05). Increased serum concentrations of CTRP3 in all intervention groups were significantly greater than in the CON group (*p* < 0.05); moreover, the increases in the RE group were significantly greater than in the ER and CON groups (*p* < 0.05). Increased serum concentrations of CTRP5 in all intervention groups were significantly greater than in the CON group (*p* < 0.05). In addition, the increases in RE were significantly greater than in COM (*p* = 0.002). Lastly, The increases in RE and ER were significantly greater than in CON (*p* < 0.05). The RE group experienced significantly greater increases in CTRP9 than all other groups (*p* < 0.05), and the decreases in serum concentrations of CRP and TNF-α were significantly greater in the RE group compared to CON and ER (*p* < 0.05).

### 3.3. Muscular Performance and Dietary Intakes

All pre- and post-intervention aerobic and strength assessment results are shown in Table 1. All three interventions showed a significantly increased VO_2max_ (RE = 6.5 mL·kg·min (95% CI = 5.4 to 7.6; *p* < 0.001; ES = 2.22), ER = 8.3 mL·kg·min (95% CI = 6.7 to 10; *p* < 0.001; ES = 2.52)), and COM = 5.2 mL·kg·min (95% CI = 3.6 to 6.8; *p <* 0.001; ES = 1.31) leg press strength (RE = 9.2 kg (95% CI = 7.7 to 10.7; *p* < 0.001; ES = 1.81), ER = 7 kg (95% CI = 5 to 8.7; *p* < 0.001; ES = 1.93), and COM = 5.4 kg (95% CI = 3.9 to 6.8; *p <* 0.001; ES = 1.5)); chest press strength (RE = 8.1 kg (95% CI = 6.5 to 9.7; *p* < 0.001; ES = 2.71), ER = 6.8 kg (95% CI = 5.5 to 8.2; *p* < 0.001; ES = 2.64), and COM = 4.5 kg (95% CI = 4 to 5; *p <* 0.001; ES = 1.98)); lower-body power (RE = 40.3 w (95% CI = 29 to 51.6; *p* < 0.001; ES = 2.14), ER = 20.8 w (95% CI = 5.4 to 36.1; *p* = 0.011; ES = 0.88), and COM = 14.2 w (95% CI = 6.6 to 21.7; *p =* 0.001; ES = 0.91)); and upper-body power (RE = 24.8 w (95% CI = 18 to 31.6; *p* < 0.001; ES = 1.92), ER = 13.3 w (95% CI = 5 to 21.6; *p* = 0.004; ES = 0.91), and COM = 16 w (95% CI = 6 to 26.1; *p =* 0.004; ES = 1.31)). The ANCOVA results indicated that the VO_2max_ values in the ER group were significantly greater than in COM (*p* = 0.009) and that all interventions resulted in higher gains compared to CON (*p* < 0.05). The increases in leg press strength, chest press strength, lower-body power, and upper-body power in the RE group were significantly greater than in the COM group, and the gains in all intervention groups were significantly greater than in the CON group (*p* < 0.05). In addition, the increases in chest press strength in the ER group were significantly greater than in COM (*p* = 0.023). The dietary intakes of participants are shown in Table 2. There were no significant changes between groups for energy (kcal/day) or macronutrient (g/day) intake from pre- to post-intervention (*p* > 0.05). 

### 3.4. Training Volume

The results shown in Table 3 depict that the main, groups, and interaction effects were significant for the training volume of RT. Additionally, there were significant differences over time in each of the groups during 12 weeks of training intervention. On the other hand, the only between-group difference was observed for the RE vs. COM groups. Regarding the training volume of ET, the data demonstrated that there was no significant difference for the groups and interaction effects, but there was for the main effect.

### 3.5. Correlations

**Figure 4:**
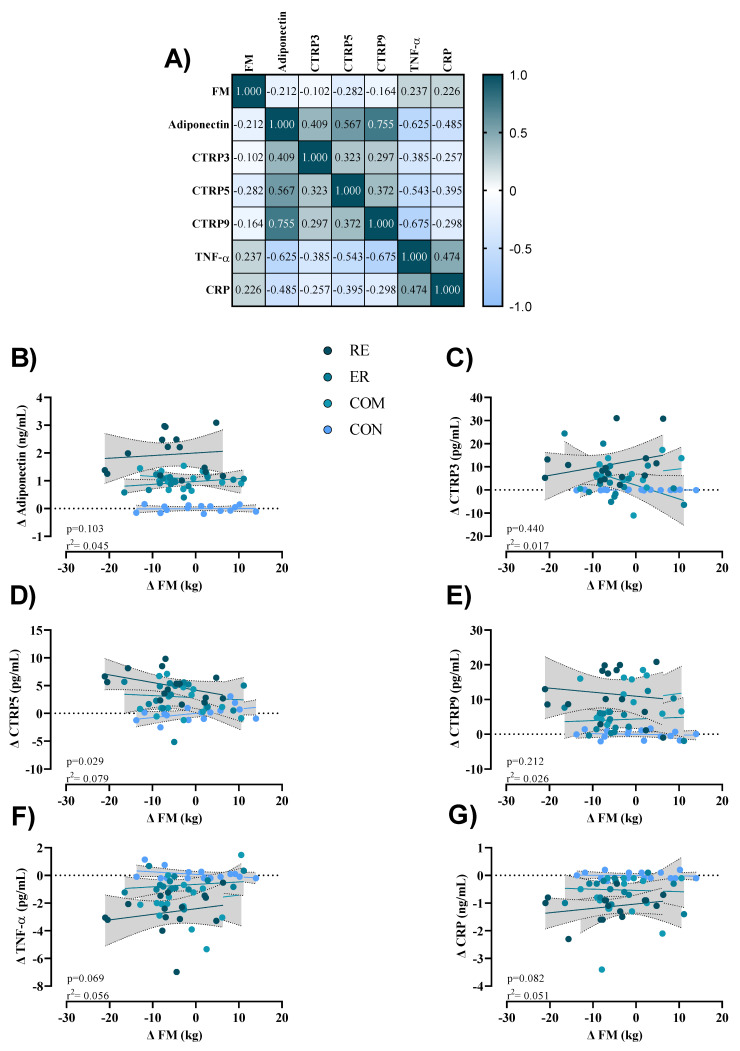
(**A**) Correlation matrix of Δ FM and serum markers (r-values as shown). The key indicates the magnitude of r (Prussian = −1 or 1, white = 0). (**B**–**G**) linear regression (Pearson’s) of Δ (serum marker) as a function of Δ FM (kg). The 95% confidence intervals are indicated by grey zones.

To assess the potential relationships between training-induced changes in FM (Δ FM) and inflammatory markers (Δ (marker)) independently of RE, ER, COM, or CON, a correlation matrix is presented in Figure 4. Serum concentrations of TNF-α and CRP showed a moderate positive relationship with Δ FM, while serum concentrations of adiponectin, CTRP3, CTRP5, and CTRP9 showed a moderate negative relationship. For linear regression of individual Δ (marker) as a function of Δ FM, the data were examined using the extra sum-of-squares F-test to first consider if pooled data could be considered as a single model. No data were considered a single group. Only Δ CTRP5 showed a significant direct linear relationship with training-induced changes in FM.

## 4. Discussion

This study sought to determine the impacts of CT order on serum concentrations of inflammatory markers (C1q/TNF-related proteins), muscle strength and power, VO_2max_, and body composition in men with obesity. Our findings indicated that CT, regardless of training order, improved inflammatory markers, body composition, muscular strength and power, and VO_2max_; however, performing RT prior to ET and combined training may prove more effective in improving CTRP3 and WHR.

The CTRPs display differential effects on regulating metabolic homeostasis and cardiovascular function [25]. Previous studies suggested that the dysregulation of CTRPs may play an important role in the pathogenesis of obesity [26,27]. Accordingly, all three CT interventions in the present study resulted in increased CTRPs; however, it should be noted that CTRP improvement in the RE group was greater compared to other intervention groups. The positive effects of various exercise strategies on CTRP have been reported in prior investigations. For example, Sadeghi et al. assessed the effects of different training protocols (RT, ET, and CT) on serum levels of CTRP5 in patients with T2DM [28]. It was determined that, similar to our findings, serum CTRP5 levels increased in all three intervention groups compared to the control. In another study, Hasegawa et al. showed that 8 w of aerobic exercise training (60–70% peak oxygen uptake for 45 min, 3 days/w) increased serum CTRP3 and CTRP5 in middle-aged and older adults [29]. Moreover, Choi et al. reported that a 3-month combined exercise program modestly increased CTRP-5 levels in obese Korean women [19]. Interestingly, they showed that exercise training significantly decreased CTRP-3 levels. Our findings showed positive effects of all training interventions on CTRP-3, CTRP-5, and CTRP-9, except for the effects of ER on CTRP-3. As a result, CTRPs may help explain a possible mechanism for the mediation of anti-inflammatory and metabolic-improving effects of exercise. It has been shown that CTRP-3 decreases IL-6 and TNF-α secretion in LPS-treated monocytic cells and suppresses nuclear factor kappa B (NF-κB) signaling [30]. Further, Wölfing et al. showed that CTRP-3 stimulates the secretion of adiponectin and resistin in adipocytes; the latter of which exert important anti-inflammatory and obesity-regulating effects, respectively [31]. In another study, CTRP-3 concentrations were positively associated with adiponectin levels following a combined aerobic and resistance training program [19]. CTRP-5 on the other hand reportedly increases glucose uptake and fatty acid oxidation in myocytes by enhancing glucose transporter GLUT-4 translocation and stimulating adenosine monophosphate-activated protein kinase (AMPK) phosphorylation, respectively [32]. Lastly, CTRP9, an adipocytokine with the highest amino acid identity to adiponectin [33], has multiple functions including regulation of glucose and lipid metabolism [33,34], acting as an anti-inflammatory agent by reducing proinflammatory cytokine expression [35,36], and prevention of cellular oxidative damage [34].

It has been claimed that adiponectin, a hormone mostly released by adipocytes, mimics many of the metabolic benefits of exercise training [37], and our results supported the findings of previous research that indicated exercise training enhances serum levels of adiponectin [14,38]. While much of this research has focused on adiponectin-elevating properties of CT [39,40], the effects of CT order on serum adiponectin are virtually unknown; only a single investigation reported on the effects of eight weeks of varied order CT, which showed no change in adiponectin in overweight women [41]. In contrast, our results showed a significant elevation in adiponectin in all exercise intervention groups (with the greatest pre- to post-change noted in the RE group), perhaps due to resistance exercise measurably stimulating adiponectin release earlier than aerobic exercise. Certainly when compared to the results of Hosseynzade et al., differences in the study population, training volume, and/or exercise intervention duration may explain such discrepancies between results [41].

It is well documented that exercise training decreases CRP and TNF-α in various populations [42,43] by inhibiting the activation of NF-κB and leading to decreased circulating concentrations of pro-inflammatory cytokines, including TNF-α and CRP [33]. Moreover, the reductions in inflammatory cytokines are reported to be directly associated with a loss in FM, particularly when exceeding 5% of overall FM [44]. Although several studies have reported the positive effects of CT on inflammation, the effects of CT order on inflammatory markers, as with adiponectin, are virtually unknown. Banitalebi et al. failed to find any differences between varied types of CT training on inflammatory markers; however, similar to adiponectin, our findings indicated that resistance exercise performed prior to aerobic exercise (RE group) may prove more effective in regulating markers of inflammation status; including CRP and TNF-α [45].

While there were no group differences between measures of upper- and lower-body strength or aerobic power and all exercise interventions (likely the result of similar training volumes), all participants in these groups showed improved chest and leg press strength, upper- and lower-body power, and VO_2max_. Similarly, our findings for body composition and anthropometrics revealed no significant between-group differences in FFM, a measure associated with strength and power; this again was likely due to similar training volumes between exercise interventions [46,47].

One of the key strengths of this study was the utilization of novel adipokines related to the CTRP family, which have not been previously evaluated following CT. Additionally, the observed correlations (mainly colorogram) between changes in FM and adipokines may yield practical insights for mitigating FM as part of health promotion efforts. The current research comes with limitations such as the fact that body composition was measured using bioelectrical impedance, a common technique that determines BFP based on methods developed for normal-weight subjects and that frequently assumes that body hydration is constant and unaffected by obesity and overweight [48]. Moreover, the gene expression of CTRPs using biopsy samples in order to more accurately characterize the upregulation of inflammatory markers was not determined but certainly provides grounds for further investigation.

In conclusion, regardless of training order, CT improved inflammatory markers, body composition, power, and VO_2max_ in middle-aged overweight and obese males. Our analysis also indicated significantly greater improvements in adiponectin, CTRP5, CTRP9, CRP, and TNF-α levels when RT preceded ET in CT sessions compared to other exercise training sequences. These findings suggested that the order of exercise training may have a significant impact on the effectiveness of CT on inflammatory markers and have potential implications for exercise prescription and optimization of health-related training outcomes. Future studies should evaluate the use of nutritional strategies (particularly protein intake) in combination with different exercise training sequences within CT along with their impact on body composition and adipokines in diverse populations and have a specific focus on females.

## Figures and Tables

**Table 1:** Descriptive statistics of variables according to group and time points.

	Group	Pre (i)	Post (j)	Diff (j − i)	*p*-Value #
Age (years)	R + E	49.96 ± 3.62	------	------	------
	E + R	51.74 ± 4.76	------	------	------
	COM	51.50 ± 4.34	------	------	------
	CON	52.63 ± 4.24	------	------	------
Height (cm)	R + E	1.71 ± 0.02	------	------	------
	E + R	1.73 ± 0.01	------	------	------
	COM	1.73 ± 0.02	------	------	------
	CON	1.72 ± 0.02	------	------	------
Body mass (kg)	R + E	93.02 ± 3.65	87.40 ± 5.32	−5.62 ± 4.20 ^d^	<0.001
	E + R	98.06 ± 4.32	93.80 ± 3.30	−4.32 ± 3.95	0.001
	COM	94.56 ± 3.61	91.25 ± 4.80	−3.31 ± 3.51	0.003
	CON	93.02 ± 3.30	92.19 ± 3.12	−0.83 ± 2.66 ^a^	0.250
BMI (kg·m^−2^)	R + E	31.68 ± 1.17	29.80 ± 1.40	−1.93 ± 1.45 ^d^	<0.001
	E + R	32.90 ± 1.50	31.50 ± 1.41	−1.44 ± 1.32	0.001
	COM	31.65± 1.43	30.54 ± 1.70	−1.11 ± 1.20	0.003
	CON	31.30± 1.10	31.01 ± 1.02	−0.30 ± 0.91 ^a^	0.256
Fat mass (kg)	R + E	40.25 ± 6.91	34.30 ± 7.70	−6.00 ± 8.30	0.014
	E + R	38.73 ± 7.00	34.64 ± 6.20	−4.10 ± 7.15	0.044
	COM	36.84 ± 7.19	33.93 ± 6.60	−3.10 ± 6.30	0.094
	CON	38.17 ± 6.80	38.35 ± 5.60	0.17 ± 8.43	0.938
FFM (kg)	R + E	52.78 ± 8.08	53.13 ± 10.80	0.35 ± 10.80	0.902
	E + R	59.33 ± 8.27	59.11 ± 5.80	−0.22 ± 7.90	0.914
	COM	57.71 ± 9.17	57.32 ± 8.25	−0.40 ± 6.71	0.822
	CON	54.85 ± 8.45	53.90 ± 7.13	−1.00 ± 8.64	0.662
WHR (m)	R + E	0.92 ± 0.02	0.89 ± 0.03	−0.03 ± 0.03 ^d^	0.001
	E + R	0.94 ± 0.02	0.92 ± 0.05	−0.02 ± 0.05 ^d^	0.185
	COM	0.92 ± 0.02	0.90 ± 0.03	−0.03 ± 0.03 ^d^	0.001
	CON	0.90 ± 0.03	0.93± 0.02	0.04 ± 0.03 ^abc^	<0.001
VO_2max_ (mL/kg/min)	R + E	20.38 ± 2.9	26.9 ± 3	6.5 ± 1.9	<0.001
	E + R	20.6 ± 2.9	29 ± 3.7	8.3 ± 2.9	<0.001
	COM	20.9 ± 3.4	26.1 ± 4.5	5.2 ± 2.8	<0.001
	CON	21.2 ± 3.1	20.5 ± 2.3	−0.6 ± 2.3	0.323
Chest press (kg)	R + E	28 ± 2.7	36.2 ± 3.2	8.1 ± 2.9	<0.001
	E + R	30.3 ± 2.8	37.2 ± 2.3	6.8 ± 2.4	<0.001
	COM	30.5 ± 2.2	35 ± 2.3	4.5 ± 0.9	<0.001
	CON	29.2 ± 3.2	29.7 ± 3.4	0.4 ± 1.5	0.372
Leg press (kg)	R + E	59.2 ± 4.8	68.4 ± 5.3	8.6 ± 2.8	<0.001
	E + R	56 ± 3.1	63 ± 4.1	6.7 ± 2.9	<0.001
	COM	57.8 ± 3.2	63.2 ± 3.8	5.2 ± 2.6	<0.001
	CON	54.2 ± 5.3	53 ± 5.8	−1.1 ± 2.6	0.122
Upper-body power (w)	R + E	337.7 ± 14.8	362.6 ± 10.9	24.8 ± 12.3	<0.001
	E + R	336.8 ±13.3	350.2 ± 15.5	13.3 ± 14.9	0.004
	COM	339.2 ± 12.3	355.3 ± 11.9	16 ± 18.2	0.004
	CON	330.7 ± 11.7	335.6 ± 10.9	4.8 ± 13.5	0.187
Lower-body power (w)	R + E	369.7 ± 18.5	410 ± 19	40.3 ± 20.3	<0.001
	E + R	376 ± 24.8	396.8 ± 22.2	20.8 ± 27.7	0.011
	COM	362.2 ± 15.2	376.4 ± 15.6	14.2 ± 13.6	0.001
	CON	378.8 ± 19.4	375.4 ± 22.4	3.4 ± 29.9	0.667

Note: the comparison between groups was performed using ANOVA. The Tukey HSD test was used to apply multiple comparisons. English letters indicate a significant difference in quantitative variables between the group in multiple comparisons (^a^ significantly different than R + E; ^b^ significantly different than E + R; ^c^ significantly different than COM; ^d^ significantly different than CON). # paired *t*-test; w, watt; kg, kilogram; m, meter; cm, centimeter; mL/kg/min, milliliter/kilogram/minute.

**Table 2:** Energy and macronutrients at baseline and during 12 weeks.

Variables	Group	Pre-Training	During Training	*p*
Energy (kcal/d)	RE	2531.2 ± 541.8	2414.2 ± 553.6	0.303
	ER	2666.7 ± 671.07	2569.8 ± 414.7	0.491
	COM	2554.2 ± 712.7	2490.3 ± 759.2	0.542
	CON	2536.1 ± 330.6	2444.1 ± 336.6	0.343
Protein (g/d)	RE	76.4 ± 17.4	75.1 ± 21.2	0.813
	ER	85.1 ± 22.3	81.3 ± 20.6	0.351
	COM	78.09 ± 27.1	72.4 ± 22.6	0.182
	CON	81.8 ± 19.9	74.6 ± 20.4	0.276
Fat (g/d)	RE	79.5 ± 24.9	78.7 ± 27.5	0.888
	ER	77.4 ± 28.6	78.3 ± 18.4	0.898
	COM	82.3 ± 36.9	84.1 ± 38.7	0.704
	CON	70.1 ± 24.7	63.1 ± 20.6	0.388
CHO (g/d)	RE	377.2 ± 89.2	351.1 ± 85.3	0.288
	ER	407.2 ± 107.02	384.8 ± 77.8	0.358
	COM	375.08 ± 91.7	360.8 ± 109.6	0.565
	CON	394.4 ± 82.9	394.3 ± 67.4	0.993

Abbreviations: CHO, carbohydrate; kcal/d, kilocalorie/day; g/d, gram/day; RE, resistance followed by endurance training; ER, endurance followed by resistance training; COM, combined resistance and endurance training; CON, control. Values are mean ± SD.

**Table 3:** Training volume of resistance and endurance training in three intervention groups.

Groups	1st–3rd Weeks	4th–6th Weeks	7th–9th Weeks	10th–12th Weeks	ANOVA Repeated Measures		
					Main Effect	Groups Effect	Interaction Effect
Training volume of resistance training (kg)							
E + R	1981.3 ± 421.5	2377.5 ± 505.8 ^a^	2971.9 ± 632.3 ^b^	2717.2 ± 578.1 ^cef^	0.001 *	0.004 **	0.001 ***
R + E ^¥^	2280.4 ± 758.2	2736.5 ± 909.8 ^a^	3420.6 ± 1137.3 ^bd^	3127.4 ± 1039.8 ^cef^			
COM	1238.8 ± 247.0	1486.6 ± 296.4 ^a^	2787.3 ± 555.9 ^bd^	2548.4 ± 508.2 ^cef^			
Training volume of endurance training (kJ)							
E + R	453.0 ± 78.2	659.0 ± 113.8 ^a^	892.4 ± 154.1 ^b^	1153.2 ± 199.2 ^cef^	0.001 *	0.922	0.922
R + E	445.1 ± 92.0	647.4 ± 133.8 ^a^	876.8 ± 181.3 ^bd^	1133.1 ± 234.3 ^cef^			
COM	460.1 ± 127.3	669.2 ± 185.1 ^a^	906.2 ± 250.7 ^bd^	1171.1 ± 324.0 ^cef^			

Main effect *; groups effect **; interaction effect ***. ^a^ Different between 4th–6th weeks to 1st–3rd weeks; ^b^ different between 7th–9th weeks to 1st–3rd weeks; ^c^ different between 10th–12th weeks to 1st–3rd weeks; ^d^ different between 7th–9th weeks to 4th–6th weeks; ^e^ different between 10th–12th weeks to 4th–6th weeks; ^f^ different between 10th–12th weeks to 7th–9th weeks; ^¥^ different between R + E vs. COM.

## Data Availability

Data sharing is applicable.
